# Incentivising research data sharing: a scoping review

**DOI:** 10.12688/wellcomeopenres.17286.2

**Published:** 2022-04-06

**Authors:** Helen Buckley Woods, Stephen Pinfield

**Affiliations:** 1 Research on Research Institute, Information School, University of Sheffield, Sheffield, South Yorkshire, S10 2TN, UK

**Keywords:** open data, data sharing, open science, research data, scoping review

## Abstract

**Background: **Numerous mechanisms exist to incentivise researchers to share their data. This scoping review aims to identify and summarise evidence of the efficacy of different interventions to promote open data practices and provide an overview of current research.

**Methods: **This scoping review is based on data identified from Web of Science and LISTA, limited from 2016 to 2021. A total of 1128 papers were screened, with 38 items being included. Items were selected if they focused on designing or evaluating an intervention or presenting an initiative to incentivise sharing. Items comprised a mixture of research papers, opinion pieces and descriptive articles.

**Results: **Seven major themes in the literature were identified: publisher/journal data sharing policies, metrics, software solutions, research data sharing agreements in general, open science ‘badges’, funder mandates, and initiatives.

**Conclusions:** A number of key messages for data sharing include: the need to build on existing cultures and practices, meeting people where they are and tailoring interventions to support them; the importance of publicising and explaining the policy/service widely; the need to have disciplinary data champions to model good practice and drive cultural change; the requirement to resource interventions properly; and the imperative to provide robust technical infrastructure and protocols, such as labelling of data sets, use of DOIs, data standards and use of data repositories.

## Introduction

The past decade has seen intensified focus on the importance of openness and transparency in research processes. Broadly characterised as ‘open science’ or ‘open research’, there are now multiple initiatives and funder/institutional policies which aim to strengthen research reproducibility, access, and utilisation through more open approaches.

Important parts of this landscape include the introduction of open access business models by publishers, the creation of open infrastructure (including networks of repositories), and development of policies supporting openness. In the area of data sharing, key initiatives include the release in 2015 of the Transparency and Openness Promotion Guidelines (TOP guidelines) produced by the Center for Open Science, and the launch in 2016 of the FAIR Principles (Findability, Accessibility, Interoperability, Reusability). Open data practices are also a part of the EU’s open science policy platform, for example in examining open data readiness in Europe ( Nagy-Rothengass, 2016).

Numerous initiatives exist to incentivise researchers to share their outputs, in the form of rewards and benefits for doing so, such as various credit and recognition schemes, or conversely, sanctions for non-compliance, for example delaying payment of a grant until compliance with a data sharing policy has been met Jubb (2016). In efforts to increase open access to research publications, approaches involving robust statements of requirements, ongoing compliance monitoring, and sanctions for non-compliance have achieved success ( Pinfield *et al*., 2014), with high levels of compliance realised with the open access policies of the Wellcome Trust and National Institutes of Health (NIH), and less compliance found with funders’ policies that have weaker or no sanctions for non-compliance ( Larivière & Sugimoto, 2018). When mandates and punitive policies appear to have such success in accelerating open access to research, this raises the question of whether a variety of similar incentives are needed to encourage open data practices?

However, there are additional complications in the area of data sharing. The effect of discipline and field may be greater in data sharing than in other open research practices ( Resnik *et al*., 2019). In addition, the deep complexity of the research system often masks the reasons why particular interventions work. Simple incentives are found to work in one discipline but not another or be unnecessary in one field and not stringent enough in the next. One suggestion is proposed by Cooper & Springer (2019) who suggest that targeting interventions at a discipline level is unfocused. They propose the idea of a ‘research data community’. This is a network of researchers drawn from any number of disciplines and fields who share and reuse data on a given topic. In designing research support interventions, they advocate detailed exploration of how researchers work with data to include trans-disciplinary knowledge areas in order to foster data sharing and scaffold existing practices, allowing the identification of “emergent data communities” where interventions can be targeted at a potentially more receptive community. Aside from the effect of discipline and field, the definition of success is also more complicated with open data interventions than with open access publishing. This is because, the data needs to be more than accessible, it needs to comply with other elements of FAIR, and where doing so may be a matter of degrees rather than absolutes (see Hardwicke *et al*., 2018, this article). Success in data sharing also depends upon alignment of the incentives and activities of multiple actors in the research system, so that practices of researchers are aligned with, for example, journal publishers’ requirements, and also in line with a funders’ policies, as reflected in the work of the National Academies of Sciences, Engineering, and Medicine (2020), to advance open science practices. Moreover, for open data sharing to be successful, it must be a truly multi-professional endeavour, with librarians, data scientists, software developers and many other professions’ expertise needed to create spaces for different types of data to be curated, shared, discoverable and reusable in an ethical and timely way ( Pasek, 2017).

The aim of this review is to identify and summarise evidence for the efficacy of known interventions and credit mechanisms to promote open data practices, to provide an overview of current research in this field. It was carried out in support of Wellcome’s role in the coronavirus disease 2019 (COVID-19) Therapeutics Accelerator (CTA), although was designed to have wider application. This review makes a particular contribution to this vast area of activity by focussing on current research describing or evaluating researcher incentives as published in the scholarly literature. In view of recent comprehensive in-depth literature reviews on open research – Jubb (2016), and Zuiderwijk *et al*. (2020) – the material in this review is limited to the last five years of publications.

## Methods

### Study design

A scoping review was chosen as most appropriate approach for this project. It can be defined as a ‘preliminary assessment of potential size and scope of available research literature. [It] aims to identify [the] nature and extent of research evidence (usually including ongoing research)’ ( Grant & Booth, 2009, p.95). The following methods were selected:

- To retrieve and characterise the literature describing interventions, incentives and credit mechanisms to elicit open sharing of interim and final research data;
- To contextualise this material within wider debates of ‘open research’ and governance measures;
- To supplement the findings from the scholarly literature with examples of such interventions provided from grey literature sources, such as the websites of policy organisations, research funders or academic publishers.

Other methodological points to note were:

- As the review was initially created in support of Wellcome’s role in the COVID-19 Therapeutics Accelerator (CTA) a protocol was not created.
- Quality assessment of evidence was not included in the review as it was completed in a short timeframe, in line with scoping review norms.

### Search strategy

A search of two key research databases was undertaken: Web of Science and LISTA (Library, Information Science and Technology Abstracts) to retrieve material to meet these requirements. No date, study or language limits were applied in the information retrieval process. In the selection process, material was limited to 2016 to 2021 publications. Initial searches took place in 2020 and were updated in June 2021. In order to complete the review in a short timeframe a pragmatic approach to the discovery of relevant materials was chosen, in order to limit the number of false positives retrieved. Terms for data (such as ‘open data’ or ‘data sharing’) were combined with terms for research actors (such as scientist* or publisher) or terms for relevant activity (such as reproducibility or reuse). These terms were tested against references from a current review ( Tenopir *et al*., 2020) to establish if it could retrieve its references. Terms for incentives were not used, with broader words and phrases being more effective. Interventions and other topics of interest were identified in the screening process within this pool of papers. An example search strategy is given in Box 1.

**Box 1. Example search strategy**

Web of Science Core Collection

# 6

#1 OR #2 OR #3 OR #4 OR #5

# 5

(TI=(("Open access") and (data)))

Indexes=SCI-EXPANDED, SSCI, A&HCI, CPCI-S, CPCI-SSH, BKCI-S, BKCI-SSH, ESCI Timespan=All years

# 4

(TI=(("Research data") and (managing or sharing)))

Indexes=SCI-EXPANDED, SSCI, A&HCI, CPCI-S, CPCI-SSH, BKCI-S, BKCI-SSH, ESCI Timespan=All years

# 3

(TI=(("Data sharing") and (publisher* or author* or publication* or funder*)))

Indexes=SCI-EXPANDED, SSCI, A&HCI, CPCI-S, CPCI-SSH, BKCI-S, BKCI-SSH, ESCI Timespan=All years

# 2

(TI=(("Data sharing" or "data-sharing" or "data-reuse" or "data reuse" or "data management" or "data-management" or "open-data" or "open data" or "data standards" or "data-standards" or "data-standard" or "data standard" or "data availability" or "data-availability") and (efficiency or reliability or reproducibility)))

Indexes=SCI-EXPANDED, SSCI, A&HCI, CPCI-S, CPCI-SSH, BKCI-S, BKCI-SSH, ESCI Timespan=All years

# 1

(TI=(("Data sharing" or "data-sharing" or "data-reuse" or "data reuse" or "data use" or "data-use" "data management" or "data-management" or "open-data" or "open data" or "data standards" or "data-standards" or "data-standard" or "data standard or "data availability" or "data-availability") and (science or scientist* or scientific or research* or academic*)))

Indexes=SCI-EXPANDED, SSCI, A&HCI, CPCI-S, CPCI-SSH, BKCI-S, BKCI-SSH, ESCI, CCR-EXPANDED, IC Timespan=All years

### Data collection

Results were transferred to Endnote (X9.3.2) where duplicates were removed. A total of 1128 results were then transferred to MS Excel (16.54) where they were screened for relevance and selected if they focused on designing or evaluating an intervention or presenting an initiative to incentivise sharing. General papers advocating the need for data sharing but without discussing specific interventions were excluded. Additional papers have been included in the references section for readers’ interest. Please see the results section for more details.

Papers in the ‘incentive’ set were further coded to record disciplinary area, context of intervention (such as funder / publisher / generic), and type of article. Due to the small number of items (38) in this set, it was not feasible to display correlations between these categories graphically, so this information is included in a narrative description.

### Presentation of results and layout of this report

The results of the review are presented below in a narrative commentary, beginning with an overview, followed by summary of different categories of incentive identified. This is followed by a summary of incentives and their outcome, before the report is concluded with a discussion of the principal messages from successful data sharing interventions. A summary table of results with key data extracted is available as extended data ( Woods & Pinfield, 2021). Please see the data availability section for access. This allows an overview of the main features of each document in one table. A full reference list of papers cited in this report, including those in the results set is presented at the end of the document. 

## Results

There are 38 items in the results set, comprising 25 research papers and 13 opinion pieces/editorials. The majority of items (20) are from scientific or medical fields, with the remaining items found within social sciences publications. None were identified from the arts and humanities. The types of interventions were varied but can be roughly classified into seven groups: publisher/journal data sharing policies, metrics, software solutions, research data sharing agreements, open science ‘badges’, funder mandates, and other initiatives. Papers concerned with academic publishing are the largest group comprising 14 papers, the next largest group being metrics with seven papers. Other categories contain ≤ five items.

**Figure 1.**
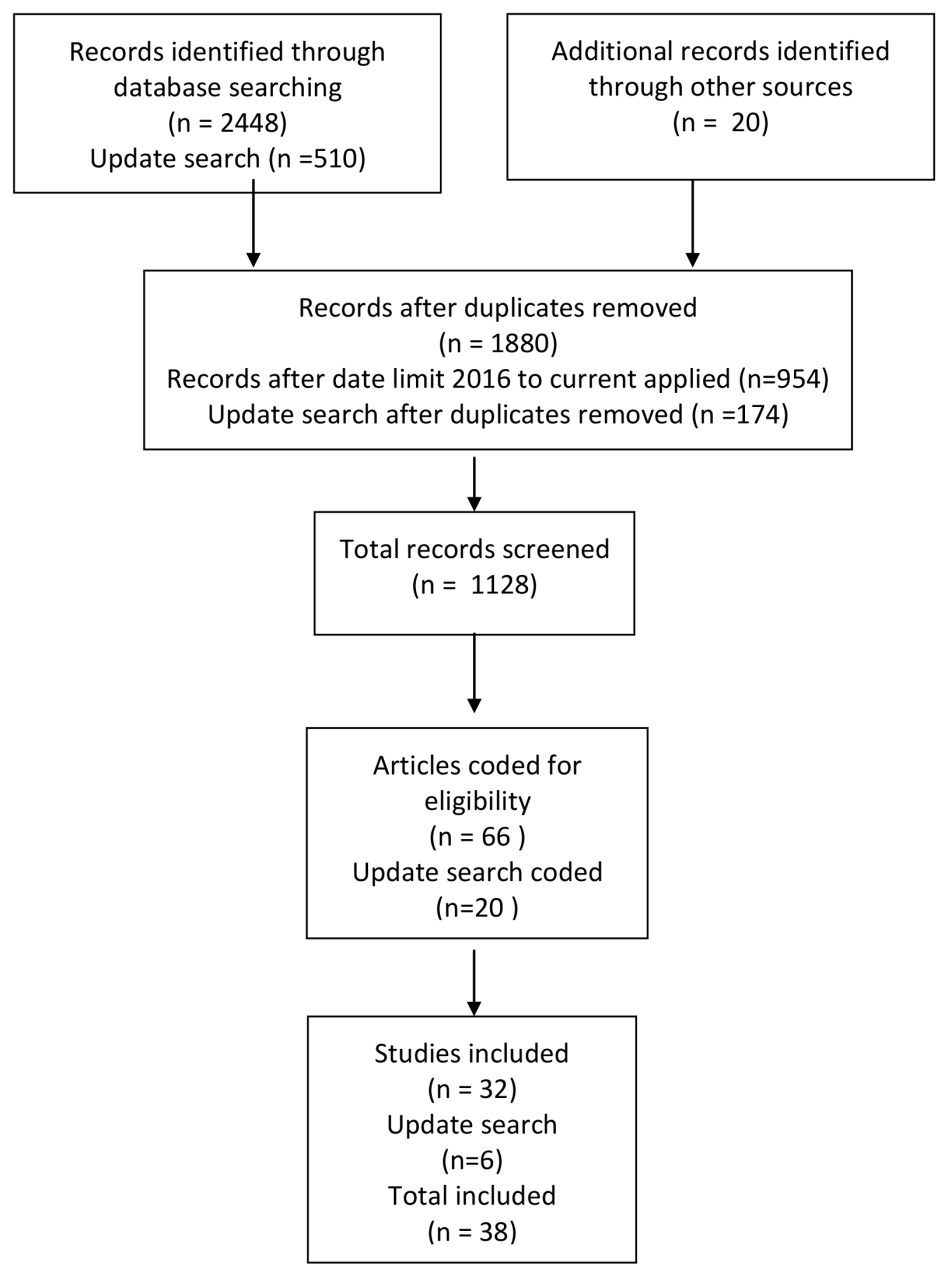
PRISMA 2009 flow diagram of the literature review.

See Figure 1 for a PRISMA (Preferred Reporting Items for Systematic Reviews and Meta-Analyses) diagram giving details of the search process.

Additional papers were selected and added to the references section for readers’ interest. These are surveys of researchers’ views linked to a related data sets presenting a correlation between views and observed behaviours: Goldstein (2018); Hickson *et al*. (2016); Kim (2017); Kim & Burns (2016); Kim & Nah (2018); Kim & Stanton (2016); Kim & Yoon (2017); Murray (2016); Staley *et al*., 2019; Yoon & Kim (2020). Likewise references that discussed the challenges of open data practices in particular geographical settings Akintola (2018); Anane-Sarpong *et al.* (2020); Jule *et al*. (2018); Kaewkungwal *et al*., 2020; Mahomed & Staunton (2021); Rappert & Bezuidenhout (2016); Reeves *et al.*, 2021; Sa & Grieco (2016); Slavnic (2017); focussed on a specific disciplinary context Barabucci *et al.* (2018); Bowman & Spence (2020); Curty *et al*. (2017); De Oliveira Carvalho (2019); De Silva & Vance (2017); Kim (2021); Krahe *et al*. (2020); Pasquetto *et al*. (2016); Poole & Garwood (2020); Rocca-Serra *et al*. (2016); Ross (2016); Thelwall *et al*. (2020); van Panhuis (2017) and Yoon & Kim (2017); or the challenges for sharing particular data types such as qualitative data Alexander *et al*. (2020); De Koning *et al*. (2019); Dilger *et al.* (2019); McLeod & O'Connor (2021); Mostern & Arksey (2016); Murillo (2018); Rasmussen (2019); Tsai *et al*., 2016; and Wutich & Bernard (2016) were included in the references section for readers’ interest.

### Publisher/journal data sharing policies

Within this theme authors investigated data sharing in academic publishing using different levels of granularity: publisher level, Castro *et al*. (2017), De Oliveira Carvalho (2016) and Federer *et al*. (2018), field / discipline specific level, Kim *et al*. (2020), Rousi & Laakso (2020), Spicer & Steinbeck (2018), Thelwall & Kousha (2017), Vasilevsky *et al*. (2017), and Wiley (2018), and interventions at the single publication level, Davies & Granhag (2019), Hardwicke *et al*. (2018), Levesque (2017), Marks (2020) and Relf & Overstreet (2021).

In the first group, **publisher level**, Castro *et al*. (2017) and De Oliveira Carvalho (2016) both take a sample of journals from the Directory of Open Access journals (DOAJ). Using a random sample from DOAJ, Castro *et al*. (2017) reports weak adoption of open data policies, beyond notable exceptions. In contrast, focusing on journals from Brazil and Portugal in science and medicine, De Oliveira Carvalho (2016) reports positively on the prevalence of open data in this context. Federer *et al*. (2018) looks at compliance with the PLOS policy on data sharing, from an analysis of 47,593 data availability statements within papers published in PLOS One, only 20% indicated compliance with the requirement to deposit data in a repository. These papers highlight the variation in compliance with policies across disciplines and fields and the variance between the formulation of a high-level policy at publisher level and its enaction at journal level.

In the **field/discipline** group, Spicer & Steinbeck (2018) investigates the field of metabolomics, and finds that a higher prevalence of published data was not correlated in journals with an open data policy. Investigating the research data sharing policies of highly cited journals in the fields of neuroscience, physics and operations research, Rousi & Laakso (2020) found a large variance in the existence, strength and content of data policies across research fields. The author highlights the need to have policies which are tailored for specific fields, for example, the treatment of particular data types and to capitalise on the existing practices of a discipline, such as the use of a repository endorsed by a research community. Vasilevsky *et al*. (2017) and Wiley (2018) both state the same aims, to investigate the ‘pervasiveness and quality of data sharing policies’ within their fields – biomedicine and engineering respectively. In a review of 318 journals’ author’s instructions and editorial policies, Vasilevsky *et al*. (2017) found that only a minority of journals (11.9%) require data sharing as a pre-condition for publication. A significant number (65%) of journals with a data sharing policy specifically made reference to reproducibility, but very few journals explicitly gave guidance on how best to make research data accessible and reusable. Wiley (2018) also analysed a sample of instructions to authors and data sharing policies, in engineering journals. Of the 28 journals analysed, the author classified 21 as ‘weak’, four as ‘strong’, with four making no reference to open data. They found no correlation between open access journals and data sharing. They also found that journals with high impact factors are not more likely to have an open data policy.

Thelwall & Kousha (2017) focus on two evolutionary biology journals that have data sharing mandates and make widespread use of a repository. They found that the data mandates were completely successful in some journals, concluding that as the major journals in the field have operated at this level of compliance since 2012, the field had transitioned into a position where data sharing had become a mainstream activity.

Kim *et al*. (2020), describe the data sharing policies of journals in life, health, and physical sciences through a sample of 700 journals indexed in the 2017 edition of Web of Science’s Journal Citation Reports. The authors selected the top journals in each quartile from the 178 categories. The policies were categorised (absent, strong, weak), and the characteristics of each journal was recorded (such as geographical location of publisher, impact factor and discipline). Regression analyses and modelling were conducted to determine whether there was a relationship between journal characteristics and the strength of the data sharing policy. Within the sample, 44% had no data sharing policy, 17.9% had weak data sharing policies, and 38.1% had strong data sharing policies (expecting or mandating data sharing). The authors report an association between certain characteristics and the strength of data sharing policies. Journals from non-commercial publishers were more likely to have no data sharing policy than those from commercial publishers. Health science journals were more likely to have no data sharing policy than life sciences journals subject area. Journals from European publishers were more likely to have a strong policy than those from North American publishers, which the authors suggest may be due to the influence of the numerous national open science initiatives in Europe. The authors conclude that these characteristics are significant factors in influencing journals’ data sharing policies. They suggest future research which takes a more nuanced approach to grading policies success, as a ‘strong’ policy requiring a data availability statement does not ultimately mean that data is shared.

This sub-theme presents a mixed picture regarding journal data policies. Authors reported the complete absence of policies, and variance in compliance where they exist. There is also variance in how authors define the strength or success of policies. In some fields, data is published regardless of the absence or presence of a policy. There appears to be a need for more detailed guidance on particular aspects of open data practices, such as how to prepare data for sharing, and how to best ensure reuse and the reproducibility of research using deposited data sets. However, where journal data sharing mandates are in place, there is evidence of widespread compliance amongst authors.

Finally, in this theme, Davies & Granhag (2019), Hardwicke *et al*. (2018), Levesque (2017), Marks (2020), and Relf & Overstreet (2021) investigate or present incentives at an **individual journal level**. The items in this sub-theme mostly present journal open data policies from different fields, which have different emphases and requirements. Davies & Granhag (2019) and Marks (2020) present editorials from the journal of *Applied Cognitive Psychology*, and the *Journal of Health Psychology,* respectively. Davies & Granhag (2019) journal has an ‘expects data’ policy with authors expected to provide a statement on data availability. Marks (2020) policy states a requirement for authors to make raw data fully available and accessible. In contrast, Levesque (2017) from the *Journal of Youth and Adolescence* provides the journal’s response to the publisher’s mandate seeking to find a balance between the benefits and costs of data sharing for authors who work with a ‘wide variety of data’. He seeks to protect authors from the ‘potential harms that can come from editors’ unilateral mandates’. In an editorial for the *Journal of the Association of Nurses in AIDS Care*, Relf & Overstreet (2021) also present open data requirements for authors, including the pre-registration of clinical trials and systematic reviews. Hardwicke *et al*. (2018) reviewed the effect of the introduction of an open data policy of the journal *Cognition* in March 2015. They conducted an interrupted time series analysis over a four-year assessment period (2014–2017). They found that the policy increased the incidence of research data being shared, and that appeared reusable. However, there were still articles without available data and with data that was not reusable when investigated. The authors point to errors such as missing values or typos or the lack of an analysis script detailing the code used to run the analyses. These papers present different disciplinary perspectives on data sharing providing an insight into the ethical challenges that accompany data sharing, particularly in some social science and humanities research.

This theme offers a variety of publisher interventions at publisher, discipline, and individual journal level. There appears to be great variance in the existence of policies and in compliance with them. There is a need for more detailed guidance for authors on how to prepare their data, tailored to the discipline or field, to increase concordance with open data policies and successful re-use of research data.

### Metrics

Overall, five papers propose or evaluate incentives associated with metrics. Bierer *et al*. (2017) suggest that ‘data authors’ should be recognised category of authorship, so people are credited through citation. In a response to this proposal, Sydes & Ashby (2017) raises the issue of accrediting work on a clinical trial and proposes the creation of a contributor database and the use of standardised terminology for people’s roles using CRediT (Contributor Roles Taxonomy). Olfson *et al.* (2017) writing in the field of clinical medicine, also propose the development of a ‘S-Index’ (sharing index) to measure data sharing and use. For each researcher who shared data ‘…publications using their shared data would be ranked in descending order by number of citations and the value of their S-index would be the number of papers (N) in this list with N or more citations’ (p. 5). The authors propose this would allow data sharing to be measured appropriately and therefore included in career progression and other activities. A call for strong public funding commitment is needed to realise this goal.

Devriendt *et al.* (2020) also propose data level metrics to credit authors for each reuse, such as downloading, data citations and so on. The assumption behind such arguments is that if people are publicly credited for their work in producing and sharing data and can therefore accrue esteem within their community for their contribution, they will be more likely to make their data open.

Mongeon *et al*. (2017) present a preliminary method to link data set creators to published authors in Web of Science in order to understand data sharing practices and contributions across disciplines. All records from Data Cite in 2015 were downloaded, these were matched with all publications identified from Web of Science in 2013–2015 using the authors names from both data sets. A large number of data set authors could be linked to authors of publications in WoS. The motivation behind the study was to gather information as a contribution toward the process of developing appropriate metrics for crediting data sharing. From the results of the study, the authors stress the importance of disciplinary differences when developing metrics for data sharing. The results found that data sharing is common in biomedical research, chemistry, medicine and biology, less so in social sciences and rare in arts and humanities. It is also not possible to share data in some fields, for example where research explores sensitive topics or uses commercial material. The authors suggest ‘any assessment of the level of data sharing must take into account what could (or should) have been shared, rather than the raw output.’ (p. 552)

Kwon & Motohashi (2020) examine the incentive of increased citation of publications that have associated with shared data. The analysed over 310,000 articles indexed in Web of Science in 2010 and comparted the number of citations of articles that shared data with those that didn’t.

They found for those articles where data was shared, citations increased in the short term but decreased over time. The authors suggest two competing factors that would affect researchers’ motivation to share their data, firstly the increased visibility of research due to data posting, but also the increased competition in the research community resulting from data sharing. Additional analysis found that the balance of these two factors changes depending upon the place of publication. In more prestigious journals the competition factor is weakened, in less prestigious journals the visibility factor is weakened using citation count data from Web of Science. Christensen *et al*. (2019) also investigated the effect of data sharing on an article’s citations. Publications in 17 high- impact journals that introduced a data sharing policy were analysed, pre and post the introduction of the policy, in a natural experiment. Where authors shared data, an increase in citations was found, but this may be linked to other factors, such as different authors or types of articles being published post policy change. The authors found no conclusive evidence that there is a link between data sharing and increased citations, but it may be one of a number of factors that led to higher citations of publications. There was no evidence as to why data sharing may increase citation rates. However, it may be one motivating factor for researchers to share their data, either in compliance with journal mandates, or as an independent practice.

A variety of interventions are proposed or evaluated in this theme, focussed on establishing mechanisms to credit authorship of research data and reward data sharers. 

The remaining categories contain fewer papers, and so summaries of the interventions below are briefer.

### Initiatives

In total, four initiatives were selected for inclusion. Hickson *et al*. (2016) present a project to encourage the use of research data management services at Griffith University, Australia. Culture change and working closely with disciplinary communities are cited as key strategies for success. Krleža-Jerić *et al*. (2016) describes the IMProving Access to Clinical Trials data (IMPACT) IMPACT Observatory and is sanguine about the progress made by the clinical trials community to open up data. The lack of data standards is cited as the main barrier to success. Pencina *et al*. (2016) present the Duke Clinical Research Institute–Bristol-Myers Squibb Supporting Open Access to Researchers Initiative. This is a service tailored to support clinical trialists. Plomp *et al*. (2019) report on a data management service tailored to disciplinary areas within Delft University of Technology, deploying ‘data champions’ and ‘data stewards’ to work with disciplinary communities. Finally, in a researcher-led initiative, Chan *et al*. (2021) describes a model for big data sharing in cell biology: a ‘data sharing trust’ which was piloted during a COVID-19 study: ‘COVID-19 Multi-Phenotyping for Effective Therapies (COMET)’ at the University of California, San Francisco (UCSF). It allows data to be generated and accessed freely by a core group of research collaborators, via a data library, with more restricted access for other institutions and the public. Data is shared in real time in both raw and processed formats.

### Badges

Rowhani-Farid *et al*. (2020) report on a randomised controlled trial to assess the effectiveness of awarding badges for data sharing in *BMJ Open*. They report that the intervention did not motivate researchers to share data and data sharing rate was low in the control and intervention group. This is in contrast to the work of the Center for Open Science (2021) on awarding badges of open science practice. Schulz (2019) reports on a conference workshop in neurochemistry. The agenda for the workshop is described, focussing on different badges designed to encourage open research, such as data sharing ‘open data badge’, pre-registering work ‘pre-registered badge’ and so on. Finally, in this theme, Hardwicke *et al*. (2021) explore the effectiveness of open badges, by assessing analytical reproducibility within 25 articles awarded open data badges in the journal *Psychological Science* between 2014–15. The study design was based on previous research reported in this review ( Hardwicke *et al*., 2018). Numerical values were reproducible without author involvement for nine articles, reproducible with author involvement for six, not fully reproducible with no author response for three, not fully reproducible with author involvement for seven articles. Unclear reporting of analytical methods is cited as the main barrier to reproducibility. The authors conclude, (reinforcing their previous findings) that the availability of data alone is not sufficient to ensure reproducibility of results.

### Software

Prado & Baranauskas (2016) looks at the effects of data sharing software using actor network theory suggesting that this provides a shared point of contact for numerous actors in the system and has potential to improve data sharing through better collaboration. Prieto *et al*. (2017) reports on ‘Shiny Tooth’ software designed to capture clinical trial data.

### Funders

Couture *et al*. (2018) report on the effectiveness of an open data policy for projects funded by the Exxon Valdez Oil Spill Trustee Council (EVOSTC). They report low compliance with this policy and only 26% of data could be recovered from 315 projects. Neylon (2017) presents an evaluation of a project to introduce data management and sharing requirements to seven projects funded by the International Development Research Center of Canada. The author concludes that the key to success greatly depends on changing research culture, not just researcher behaviour. Rollando *et al*. (2020) report on a survey to establish the number of French clinical trials funders who have a data sharing policy. Using an online survey, 190 funders were contacted, with 94 failing to respond and 65 excluded as not eligible (not funders of clinical trials) with 31 funders included, only nine (29%) had enacted a data-sharing policy.

Finally, also in the field of clinical trials, Gaba *et al*. (2020) assess the compliance of funded randomised controlled trials (RCTs) with data-sharing policies of commercial and non-commercial funders in the years 2016–2018. Under half of those funders surveyed had a data sharing policy, with a subset of the policies mandating data sharing. Two random samples of 100 RCTs registered on Clinicaltrial.gov funded by those with a data-sharing policy found good coverage of data-sharing statements (77 non-commercially funded; 81 commercially funded RCTs), with an intention to share data made in a small number of trials (12% non-commercial, 59% commercial). The authors suggest as a first step towards greater consistency in data sharing practices across RCT, a collated, comprehensive and updated list of funders’ policies should be created in order to work towards standardisation of such policies. A lack of incentives for researchers to comply with policies could also limit their success.

### Research data sharing agreements (generic)

Mueller-Langer & Andreoli-Versbach (2018) reports on the unintended negative consequences of data sharing agreements, such as researchers delaying sharing their data in order to fully exploit its potential in their own continuing research before publishing it. Pasek (2017) located at the University of Wyoming, describes an evaluation of government data sharing policies for US government research grants. The policies have limited success, but through this evaluation a tailored research data management service is being developed to fill the gaps in the policy guidance. Finally, Polanin & Terzian (2019) report on a randomised controlled trial investigating the effects of data sharing agreements on researchers’ willingness to share individual participant data. This study focussed on primary study authors whose studies were included in meta-analysis in the social sciences. Through searches of bibliographic databases 1,207 authors were invited to participate in the study, with 580 (48.1%) allocated randomly to the intervention group (where participants received a hypothetical data-sharing agreement), and 627 (51.9%) to the control group (where participants did not receive the data-sharing agreement). Confounding factors were controlled for using numerous measures. The study found that participants who received the data-sharing agreement were more willing to share their data set (24% more likely) compared to those in the non-intervention group. See Table 1 for a summary list of interventions from the included study.

**Table 1.** Summary list of interventions from included studies.

Study	What is the incentive?	Did it succeed and why?
Castro *et al.* (2017)	Data sharing policies of open access journals.	No, weak adoption beyond notable exceptions.
Chan *et al.* (2021)	‘Data sharing trust’ for researchers in a collaborative scientific project sharing big data sets.	Yes, technical and governance infrastructure in place; allows for differential levels of access to data; aligned with researchers’ incentives.
Gaba *et al.* (2020)	Evaluation of data sharing policies and compliance with commercial and non-commercial RCT funders.	No, sub-optimal coverage of policies and limited implementation.
De Oliveira Carvalho (2016)	Practices of open access journals from Brazil and Portugal in science and medicine.	Yes, reasons not reported in abstract. Full text not available in English.
Federer *et al.* (2018)	Compliance with the PLOS policy on data sharing.	No, only 20% compliance.
Spicer & Steinbeck (2018)	Open data sharing in metabolomics.	No, no relationship detected.
Rollando *et al.* (2020)	Survey to establish the number of French clinical trials funders who have a data sharing policy.	Description only.
Rousi & Laakso (2020)	Open data sharing in neuroscience, physics and operations research.	Inconclusive, mixed picture, some fields do not have policies in place.
Vasilevsky *et al.* (2017)	Data sharing policies in biomedical literature.	No, only a minority of journals require data sharing as a pre-condition for publication.
Wiley, 2018	Data sharing policies in engineering	No, they found no correlation between open access journals and data sharing.
Thelwall & Kousha (2017)	Data sharing mandates of two evolutionary biology journals.	Yes, data sharing has become a mainstream activity in this field.
Davies & Granhag (2019); Levesque (2017); Marks (2020)	Journal open data policies.	Publication of open data statement only.
Hardwicke *et al.*, 2018	Journal Open data policy.	Yes, increased the amount of data shared.
Hardwicke *et al.*, 2021	Open data badges.	No, values presented in the articles sampled were reproducible without author involvement for only 36% of articles.
Bierer *et al.* (2017)	Data authors.	Proposal only.
Sydes & Ashby (2017)	Accrediting contributions on a clinical trial.	Proposal only.
Devriendt *et al.* (2020)	Data level metrics to credit data authors.	Proposal only.
Kwon & Motohashi (2020)	Increased citations as an incentive for data sharing.	Yes, citations increased in the short term but decreased over time.
Christensen *et al.* (2019)	Increased citations as an incentive for data sharing.	Possibly, may be one of a range of factors linked to increased citation rates. No evidence as to why sharing data may increase citation rates or if it incentivises data sharing.
Hickson *et al.* (2016)	Culture change.	Yes, identified ‘attitude’ as the key attribute to focus on in the refinement of library services for research data management.
Kim *et al*. (2020)	Journals data sharing policies in life, health, and physical sciences.	Description only.
Krleža-Jerić *et al.* (2016)	IMProving Access to Clinical Trials data (IMPACT) IMPACT Observatory.	Some promising early results. Main barriers to success are the lack of data sharing standards.
Mongeon *et al.* (2017)	A preliminary method to link data set creators (from Datacite), to published authors in Web of Science in order to understand data sharing practices and contributions across disciplines.	Exploratory only.
Olfson *et al.* (2017)	Proposal of the ‘S-Index’ (sharing index) to measure the number and impact of peer reviewed publications in which researchers have shared their data with other research groups.	Proposal only.
Neylon (2017)	Introduction of research funder data management and sharing requirements.	Yes, key finding is the importance of changing research culture, not just researcher behaviour.
Pencina *et al.* (2016)	The Duke Clinical Research Institute–Bristol-Myers Squibb Supporting Open Access to Researchers Initiative.	Not reported
Plomp *et al.* (2019)	Data management service tailored to disciplinary areas within Delft University of Technology.	Yes, established that working with individual communities is essential. Success is limited by the need for changes in the academic reward system.
Polanin & Terzian (2019)	Data sharing agreement to encourage sharing of individual participant data.	Yes, those that received the intervention (a hypothetical data-sharing agreement) were 24% more likely to share their data than those in the control group (who did not receive the data-sharing agreement).
Relf & Overstreet (2021)	Journal open data policy.	Not reported.
Rowhani-Farid *et al.* (2020)	Badges (in BMJ Open).	No, did not motivate researchers to share data.
Schulz (2019)	Workshop on open science badges.	Not reported.
Prado & Baranauskas (2016)	Data sharing software (in general).	Inconclusive.
Prieto *et al.* (2017)	Shiny Tooth software to capture clinical trial data.	Not reported.
Couture *et al.* (2018)	Funder mandate.	No, low compliance.
Mueller-Langer & Andreoli-Versbach (2018)	Generic data sharing policies.	No, may produce unintended negative consequences, such as delaying release of data.
Pasek (2017)	Government data sharing policies for US government research grants.	Limited success, a tailored service is being developed to complement the guidance given in government policy for research data management.

## Discussion and study limitations

### Limitations of the scoping review process

Scoping reviews are designed to provide a quick response to identify the ideas or interventions that have been published on a particular topic. As Tricco *et al*. (2016) suggest, this type of review is limited in its very nature, as it aims to provide breadth rather than depth of information. As in the case of this report, scoping reviews are often initiated as part of a wider project, to inform primary research or identify gaps in the literature. As highlighted by Grant & Booth (2009) when read in isolation, prudence should be exercised in the interpretation of the findings as quality assessment methods are not usually applied in a scoping review, as is the case in this review.

### Key messages

Of the 38 interventions listed above, 10 reported some degree of success. The key messages from these papers are presented below.

*
**1. **
*  ***There is a need for clear data sharing agreements, strong governance, and good technical infrastructure***

Chan *et al*. (2021) found success in a pilot researcher-led initiative to share large data sets within a COVID-19 research collaboration in cell biology (COMET) based on building a “data sharing trust” amongst actors. The **key factors for success** were:

- an existing institutional data sharing platform was used
- a data sharing agreement was put in place for the project
- the COMET project executive committee monitored the pilot and intervened where necessary to resolve problems.

The data sharing agreement allowed for all researchers to see the data, but permission had to be gained from the owner of the data to reuse it. In addition, authorship was offered to the team / lead investigators who generated the data initially. The agreement provided protection against being ‘scooped’ and rewarded data generation and sharing. Another notable success factor was that the Comet project executive committee monitored the pilot and intervened where necessary to resolve problems. For example, they assigned additional personnel for project and data management to streamline the data sharing process and resolved conflicts where different groups began working on similar or overlapping ideas.

*
**2. **
*  ***Data sharing agreements work and can be optimised by addressing authors’ a priori concerns about data sharing***

Polanin & Terzian (2019) found evidence to support their hypothesis that **a data-sharing agreement affects authors attitudes and willingness to share** individual participant data to be included in meta-analyses. Authors concerns can also be addressed in advance through a data-sharing agreement, increasing the success of this intervention. Authors primary concerns identified in the study were: the need for adequate storage and accessibility of data; the limits of reuse once shared; the time taken to prepare the data for sharing, and the right to contribute to the meta-analysis that their data would be included within. The key message is that when seeking data from primary study authors, meta-analysts should send a data-sharing agreement, which addresses authors key concerns, in addition to an email asking for the data set.

*
**3. **
*  ***Credit and competition effects of data sharing need to be recognised and incorporated in policies***

Kwon & Motohashi (2020) highlight the need to address two factors when creating a data sharing policy: **to harness the benefits of increased citations as a motivator for researchers to share data, and to mitigate the deleterious effects of this practice, namely increased competition**. They make two recommendations, firstly increased legal protection for the owners of research data, enabling researchers greater control of who accesses and uses their data, possibly using a licensing scheme. This may be too complicated to realise in practice, but if practicable would address one significant disincentive to data sharing. The second recommendation is to mandate that all researchers disclose their data, possibly as a condition of receiving public funds. The authors concede that this policy may also result in researchers undermining this measure by not curating their data appropriately for sharing.

*
**4. **
*  ***Journal data sharing policy find success using existing disciplinary infrastructures and building on existing behaviours***

Thelwall & Kousha (2017) found that data sharing mandates were highly successful in evolutionary biology journals that had signed up to a ‘Joint Data Archiving Policy’ (JDAP) datadryad.org/pages/. These mandates have been in place since 2012 and data sharing has become a mainstream activity. The reason for success is not stated explicitly, but **the effectiveness of the policy may lie in its joined-up approach across a field** with several journals signing up to the policy.

The data was held at an existing digital repository (Dryad) designed and used for evolutionary biology research data, so linking to this existing resource meant more chance of success. It had already proved to be fit for purpose for this particular type of data, and people were already using it, so new habits did not have to be formed and there was no additional time to spend learning how to use new software to deposit the data.

It was also set up so that authors received automated instructions on how to submit their data to the repository from the journal they would publish in.

*
**5. **
*  ***Information service providers are advised to focus on changing the attitudes of their users by providing individually tailored support***

This project ( Hickson *et al*., 2016) aimed to improve adherence to the use of data management processes by researchers in an Australian University. To plan a successful behaviour change strategy, they surveyed researchers and interpreted the data based on the ACOMB behaviour change model. With Attitude (A) influencing C. Capability; O. Opportunity; and M. Motivation, all of which interact to generate behaviour (B).

**The authors recommend** **that for an intervention to be successful ‘attitude’ is the key element to focus on changing**, as it is the main barrier to good data management practices. To this end, interventions were designed to meet individual’s capabilities and needs to affect attitudes and promote the use of safe and secure institutional data management services.

*
**6. **
*  ***Librarians have a key role in bridging policy and practice***

Pasek (2017) examined government data sharing policies for US government research grants, focusing on the data sharing policy of the National Science Foundation. The author states several shortfalls in the policy including undefined terms, ambiguous definitions, with minimal guidance and examples of data management plans provided for users. **The author suggests that librarians are best placed to bridge the gap between the policy and its implementation** by researchers by supporting grantees to practically create data management plans (DMPs), provide technical support to curate and share data, and provide expertise in metadata and data management standards, as well as expert knowledge of open data and open access initiatives.

*
**7. **
*  ***Data sharing interventions by research funders should aim to change research culture not just researcher behaviour***

In an intervention to introduce data management and sharing requirements for award holders from a funding organisation, Neylon (2017) stresses the importance of changing research culture, not just researcher behaviour. This finding points to the adoption of longer-term policy goals and **five recommendations are given for policy formulation:**

- The two functions of a policy: first, the message that something is an important issue (such as data management), and second, the steps to change people’s behaviour, need to be in concert and mutually reinforcing.
- The message feature of a policy is important and will work even better if it goes with the grain of existing feelings or thinking on a topic.
- The policy needs to make sense to those within the funding organisation, inspiring individuals, cohering with, and enlivening the organisation’s culture and values. It should empower people to act and provide the necessary resources and infrastructure for those managing its implementation.
- Staff time (particularly Program Officers) and resources need to be made available to ensure that the grantees are supported to understand and adhere to the policy and that adherence is monitored and followed up. Otherwise, the message to researchers is that data management is not important after all.
- Staff time and resources need to be a long-term commitment to ensure policy success. Through continued practical commitment to implementation, adoption of the policies will be more widespread with greater numbers of people changing their behaviour, to eventually become mainstream behaviour. Grantees who engage with these new ways of working become part of a community driving best practice, and through the visibility of their actions encourage others to join them. This advocacy role rewards researchers, creates more visibility and publicity for these practices (and underlying policy) and creates a virtuous circle, attracting more people to join in.

*
**8. **
*  ***Journal open data policy compliance needs to be overseen by designated staff, and better data storage and labelling are required***

Hardwicke *et al*., 2018 found that **the rate of data being shared in the journal they examined (** *Cognition* **) had increased through an open data policy**. An interrupted time-series analysis found data availability statements had increased from 25% to 78% after the policy had been introduced. They found that the amount of data that was reusable moved from 22% to 62% after the policy was introduced. The reason why the policy worked is not explored but whether it worked beyond face value. The authors conducted several exploratory analyses accessing and repeating research methods described in *Cognition* articles. **Author’s suggestions resulting from their analysis:**

- Policies need to be consistently enforced to ensure data is available and reusable.
- Offer clear guidelines for authors on data management including checklists to ensure procedures are followed.
- Assign a specific member of an editorial team to oversee data assessment and policy compliance.
- Journals need to provide clearer labelling of additional files, to describe exactly what they contain. Avoid bland, time-wasting titles like ‘supplementary data’.
- More consistent licensing is needed, so that it is clear if a data set can be reused and there is no uncertainty about this.
- Use of repositories instead of journals own ‘supplementary materials sections’ to avoid broken links to information. Repositories future proof materials by creating read only, time stamped files that have DOIs and can therefore be cited easily.

*
**9. **
*  ***To increase clinical trials data sharing influential organisations’ declarations and policies make a difference, and the use of persistent identifiers are essential***

Krleža-Jerić *et al*. (2016) report on the IMProving Access to Clinical Trials data (IMPACT) Observatory. It has numerous goals, only one of which is to monitor data sharing initiatives and assess their impact. **Preliminary findings on initiatives suggest:**

- Statements and declarations from influential organisations have contributed to an increase in data sharing such as ICMJE, Ottawa statement, WHO, Cochrane, Declaration of Helsinki, the REWARD (REduce research Waste And Reward Diligence) Campaign, the Institute of Medicine report (IOM), and the AllTrials initiative (14,20-25).
- Regulators are also important players in increasing open data practices such as the European Medicine Agency (EMA) 2014 policy on data sharing ‘and its consequent actions sharing the clinical study reports’
- In order to cite data a persistent identifier should be assigned e.g. DOIs
- Data sharing standards and lack of data sharing platforms (in comparison to resources like PubMed and Web of Science for discover of scholarly literature) remain challenges.

*
**10. **
*  ***Small changes matter, working with disciplinary communities is essential and resourcing the service is necessary***

Plomp *et al*. (2019) reports on a data management service tailored to disciplinary areas within Delft University of Technology. The authors advocate pursuing interventions in data management, even though success will be limited due to systemic problems within the academic reward system. **The key findings on delivering a successful intervention** are to work with individual disciplinary communities and have a dedicated member of staff (a ‘data steward’) who has expertise in data management within the subject, which includes first-hand knowledge of conducting research in a relevant subject area through a doctoral qualification. The role of academic data ‘champions’ was also highlighted, academic staff who model good practice and advise peers on data management. In conclusion, data stewards drive cultural change enabled by a suitable technical infrastructure, and their understanding of existing cultural norms and ways of working in different disciplinary areas.

### Summary

In summary, the key ‘take home’ points from the studies are:

- The need to build on existing cultures and practices, meeting people where they are and tailoring interventions to support them,
- The importance of publicising and explaining the policy/service widely,
- The need to have disciplinary data champions to model good practice and drive cultural change,
- The requirement to resource interventions properly,
- The imperative to provide robust technical infrastructure and protocols, such as labelling of data sets, use of DOIs, data standards and use of data repositories.

Whilst these studies all focus on particular contexts and actor groups, it is reasonable to assume that many of the insights they gain are transferable to other situations, although the extent of transferability will vary depending on a complex set of factors.

## Conclusion

This scoping review of incentives and credit mechanisms for open data sharing is based on data identified from Web of Science and LISTA, limited from 2016 to 2021. A total of 1128 papers were screened, with 38 items being included. These items comprised a mixture of research papers, opinion pieces and descriptive articles. The material was categorised into seven groups according to intervention: publisher/journal data sharing policies, metrics, software solutions, research data sharing agreements in general, open science ‘badges’, funder mandates, and initiatives.

The material in this review does not reveal any new types of incentive or credit mechanism, nor do we claim to have identified any panaceas. However, the evidence that is included is taken from many different contexts, disciplines and perspectives, and illustrates a range of activities and experiments. As such, this set of material reflects the complexity of the open data movement and the different success levels and approaches to open data sharing that exist across the disciplines. With numerous incentives being trialled within individual sectors of the research system, it seems that the cutting edge of the movement is now investigating aligned incentives as the most beneficial way forward ( National Academies of Sciences, Engineering and Medicine, 2020). The evidence in this review also suggests (in line with previous evidence), that tailored incentives, bespoke to particular disciplines and fields, that harness existing working practices, working within developing community cultures and are appropriately resourced are more likely to be successful.

## Data availability

### Underlying data

All data underlying the results are available as part of the article and no additional source data are required.

### Extended data

ORDA (Figshare): Summary of data. Incentivising data sharing: a scoping review.

ORDA Repository. https://doi.org/10.15131/shef.data.16874422 ( Woods & Pinfield, 2021).

Data are available under the terms of the Creative Commons Zero "No rights reserved" data waiver (CC0 1.0 Public domain dedication).
