# Supplementary material for: Incentivising research data sharing: a scoping review
Source: Wellcome Open Res. 2022 Apr 6;6:355. Originally published 2021 Dec 21. [Version 2] doi: 10.12688/wellcomeopenres.17286.2 (PMC8808319; doi:10.12688/wellcomeopenres.17286.2)
Supplement: Peer review history

# Peer review: Reviewer response for version 2

**DOI:** 10.21956/wellcomeopenres.19716.r49791

**Published:** 2022-04-14

**Authors:** Birgit Schmidt

**Affiliations:** 1 Göttingen State and University Library, University of Göttingen, Göttingen, Germany

Thank you for your kind response and your careful revisions, all looks very good and I have no further comments. Best wishes!

Are the rationale for, and objectives of, the Systematic Review clearly stated?

Yes

Is the statistical analysis and its interpretation appropriate?

Not applicable

Are sufficient details of the methods and analysis provided to allow replication by others?

Partly

Are the conclusions drawn adequately supported by the results presented in the review?

Yes

Reviewer Expertise:

Open Science, research data management, reproducibility

I confirm that I have read this submission and believe that I have an appropriate level of expertise to confirm that it is of an acceptable scientific standard.

# Peer review: Reviewer response for version 1

**DOI:** 10.21956/wellcomeopenres.19111.r47795

**Published:** 2022-02-03

**Authors:** Birgit Schmidt

**Affiliations:** 1 Göttingen State and University Library, University of Göttingen, Göttingen, Germany

The review provides a comprehensive overview of mechanisms and evidence of their effectiveness for incentivizing researchers to share their data. The motivation and the objectives of the review are clearly stated. However, it was a bit surprising that the authors seem to consider policies as such as incentives for researchers to share data, without further discussing their definition and scope of incentives in the introduction. In an earlier review, Rownhani-Farid *et al.* (2017) ^1^ have decided to exclude policies as studies had observed that there was only low uptake in the considered field and a lack of rewards to researchers for sharing their data.

 The study design and the overall search and selection strategy are well described. The analysis is very valuable as it covers a broad range of interventions and provides sufficient detail on the results and effectiveness from the studies and other materials included in the review (further details are provided in an openly available dataset).

 However, reproduction or replication would be impossible as the authors do not specify their search strategy in detail. Primarily, they provide search string examples (Box 1, p. 4), without stating which string was finally used for their initial search and the updated search (Figure 1, p. 5). In addition, it remains somewhat unclear if the retrieval was generally restricted to the title of the work, or targeted the topic (i.e. covered the title, abstract and keywords). Regarding the search strings used, it would have been good to explain why the truncation publisher* and not publish* was used (why is the focus on publisher and not that data is published?).

 Other comments:

- Although research performing organisations are to some degree covered, it remains unclear why they are not mentioned in the Methods section (e.g. p. 3).
- Consider archiving the dataset in an open format (e.g. csv), together with a README file with basic documentation. The first column/variable contains more than one value (i.e. the data does not follow the tidy data principles), and one row uses a reference that should be made explicit (“the above article”).

The conclusions drawn by the authors are well supported by the results presented in the review. However, some statements describing the overall context of the review in the introduction would benefit from references, e.g.

- Consider to add further references to statements in the introduction (p. 3, “Numerous initiatives exist to incentivize researchers…”, “delaying payment of a grant until compliance with a data sharing policy has been met”). Other statements seem to anticipate findings of the review (p. 3, “Simple incentives seem to work in one discipline but not another”).
- Complete the missing reference to Larivière & Sugimoto (2018), ^2^ it is provided in the text but not linked and listed in the references.

Other possible errors:

- Some references are by multiple authors but are referred to as single author (e.g. p.5, “Castro *et al.* (2017) reports… “).
- Typo in the table on p. 10: “datarelf” = ?
- Formatting on p.12: One sentence within key message 8 is formatted as a sub-section heading (“Author’s suggestions on resulting from their analysis:”).

Are the rationale for, and objectives of, the Systematic Review clearly stated?

Yes

Is the statistical analysis and its interpretation appropriate?

Not applicable

Are sufficient details of the methods and analysis provided to allow replication by others?

Partly

Are the conclusions drawn adequately supported by the results presented in the review?

Yes

Reviewer Expertise:

Open Science, research data management, reproducibility

I confirm that I have read this submission and believe that I have an appropriate level of expertise to confirm that it is of an acceptable scientific standard.

## References

[1] : Erratum to: What incentives increase data sharing in health and medical research? A systematic review. *Res Integr Peer Rev* .2017;2: 10.1186/s41073-017-0034-y 7 10.1186/s41073-017-0034-y 29457799PMC5803620

[2] : Do authors comply when funders enforce open access to research?. *Nature* .2018;562(7728) : 10.1038/d41586-018-07101-w 483-486 10.1038/d41586-018-07101-w 30356205

# Response

**Published:** 2022-03-23

**Authors:** Helen B Woods

Thank you for reviewing our paper. Please see below for our responses to each point in your helpful and thorough review.

 With reference to the inclusion of policies in our review. In line with our original brief for the project, we interpreted the idea of an incentive broadly, to include any type of incentive or credit mechanism, irrespective of where they emanated from. 

 Regarding the search strategy. All the search strings were used, so we have made this clearer by adding Boolean ‘OR’ between the search sets. At the beginning of each search string ‘TI’ indicates the title field, in Web of Science. Various terms for different actors in the research system were used in the search sets, such as author or scientist, ‘publisher’ is another of these terms. 

 With reference to the ‘other comments’ and ‘other possible errors’ section of your review. Regarding the methods section, we did include search terms for actors who conduct research such as 'research*' or 'academic*' or 'scientist*' and so on. Regarding the ‘summary of data table’, we have edited this table and it now has only one value in the first column. The text ‘the above article’ has been removed and replaced by the relevant reference. Typographical errors have been addressed. The file has been saved as a CSV file and uploaded to the University of Sheffield research data repository (ORDA) replacing the initial file, with an additional README file also uploaded. We have added an additional appropriate citation in the introduction. The typographical and formatting errors you raised have been addressed and both tables reviewed for such errors.****

# Peer review: Reviewer response for version 1

**DOI:** 10.21956/wellcomeopenres.19111.r47798

**Published:** 2022-02-01

**Authors:** Carol Tenopir

**Affiliations:** 1 School of Information Sciences, University of Tennessee, Knoxville, TN, USA

This is a complete review of the literature on why researchers share (or do not share) their research data and incentives to sharing. Over 1100 papers were reviewed to develop seven themes that are relevant to why researchers share data. Publishers, funders, and data managers will be especially interested in the findings that look at how policies, metrics, software, agreements, badges, mandates and other initiatives influence sharing of research data.

 The two main limitations of the paper are clearly stated and acknowledged. The first is the choice of Web of Science and LISTA as the source of the papers being reviewed. This perhaps is the reason for the second limitation--that is the data sharing incentives and discussions are almost all regarding scientific or medial data, with some referencing social science data. Results cannot then be extrapolated to humanities data or scholars of the humanities.

 These are not major limitations and, since even the definition of data may differ between science and humanities, is more likely a strength that allow robust conclusions.

Are the rationale for, and objectives of, the Systematic Review clearly stated?

Yes

Is the statistical analysis and its interpretation appropriate?

Not applicable

Are sufficient details of the methods and analysis provided to allow replication by others?

Yes

Are the conclusions drawn adequately supported by the results presented in the review?

Yes

Reviewer Expertise:

Information sciences; publishing behaviors; research data behaviors

I confirm that I have read this submission and believe that I have an appropriate level of expertise to confirm that it is of an acceptable scientific standard.

# Response

**Published:** 2022-03-23

**Authors:** Helen B Woods

Thank you for reviewing our paper. 

 Thank you for your thought provoking comments regarding data sharing across disciplines. Your comments suggest both conceptual and technical implications for reviews of this topic. Firstly, the fact that definitions of data differ between disciplines and indeed terms such as ‘data’ and ‘methods’ may not be used in humanities fields, particularly theoretical areas. Secondly, in order to broaden a review of this type to wider disciplinary areas this concept would need to be translated into search terms that could capture uses of ‘data’ in the humanities. This was not considered at the outset of the review as It was carried out in support of Wellcome’s role in the coronavirus disease 2019 (COVID-19) Therapeutics Accelerator (CTA). We think there is also an interesting discussion about how far humanities researchers would welcome the terms ‘data’ and ‘methods’ and other language drawn from empirical research to be ascribed to their work. This would probably be a mixed-picture, depending upon field and type of knowledge being produced. 

 With reference to the databases used in this project, we searched the WoS Core Collection and included the AHCI and other humanities sources. Where cross-disciplinary interventions met our inclusion criteria these were included in the review such as Plomp et al (2019). We also included additional citations that were beyond the scope of our review question, but may be of interest to our readers. These include papers that address data sharing through a focus on (respectively) researchers’ views, geographical area, field, and type of data.

# Peer review: Reviewer response for version 1

**DOI:** 10.21956/wellcomeopenres.19111.r47797

**Published:** 2022-01-18

**Authors:** Cameron Neylon

**Affiliations:** 1 Centre for Culture and Technology, Curtin University, Perth, WA, Australia

This report provides a scoping review of approaches to encourage research data sharing. It provides an initial literature list of 38 core items that examine or introduce interventions, approaches and evaluations for data sharing programs.

 It provides a broad thematic analysis and an initial synthesis of the literature identified. It also provides some level of taxonomy of interventions that would be interesting to see developed further in the future.

 One of the issues with an approach like this is that it is necessarily scoped down on specific databases. Nonetheless, one piece of work I think would enrich this analysis is the strand of work from Cooper and Springer of Ithaka S+R ^1^^,^^2^ which emphasises the role of communities (I would use the word culture, but there is a close alignment) which to me pulls the conclusion in a slightly different direction, that the cutting edge is not so many incentives (at least in the sense of individual micro-economic interests) as to how to shift culture so that incentives follow. Arguably this is a fine semantic distinction and the authors may of course disagree!

 This report provides a useful and valuable summarisation and initial synthesis of an emerging and dynamic literature. Perhaps it is also worth a comment on how apparently small this core literature is? For an area that has defined the focus of policy makers for nearly a decade, it seems surprising that there are only 38 substantive studies or interventions actually examining what works!

Are the rationale for, and objectives of, the Systematic Review clearly stated?

Yes

Is the statistical analysis and its interpretation appropriate?

Not applicable

Are sufficient details of the methods and analysis provided to allow replication by others?

Yes

Are the conclusions drawn adequately supported by the results presented in the review?

Yes

Reviewer Expertise:

Research evaluation and policy, open research, open access, data sharing

I confirm that I have read this submission and believe that I have an appropriate level of expertise to confirm that it is of an acceptable scientific standard.

## References

[1] : Data Communities: A New Model for Supporting STEM Data Sharing.2019; 10.18665/sr.311396 10.18665/sr.311396

[2] : Data Communities: Empowering Researcher-Driven Data Sharing in the Sciences. *International Journal of Digital Curation* .1970;15(1) : 10.2218/ijdc.v15i1.695 10.2218/ijdc.v15i1.695

# Response

**Published:** 2022-03-23

**Authors:** Helen B Woods

Thank you for reviewing our paper. 

 Thank you for alerting us to Cooper and Springer’s interesting work on research data communities. We agree this is important as it provides a close up examination of multi-disciplinary research practices and the implications for data sharing springing from this type of knowledge production. 

 With this in mind we have made reference to this work in the introduction section and we have made a minor change to the conclusion to reflect this different emphasis.
